# Climate change and health: rethinking public health messaging for wildfire smoke and extreme heat co-exposures

**DOI:** 10.3389/fpubh.2024.1324662

**Published:** 2024-03-25

**Authors:** Eric S. Coker, Susan Lyon Stone, Erin McTigue, Jiayun Angela Yao, Emily P. Brigham, Michael Schwandt, Sarah B. Henderson

**Affiliations:** ^1^Environmental Health Services, British Columbia Centre for Disease Control, Vancouver, BC, Canada; ^2^Office of Air Quality Planning and Standards, United States Environmental Protection Agency, Research Triangle Park, NC, United States; ^3^Air and Radiation Division, United States Environmental Protection Agency, Region, Seattle, WA, United States; ^4^Division of Respiratory Medicine, Department of Medicine, University of British Columbia, Vancouver, BC, Canada; ^5^Vancouver Coastal Health Research Institute, Vancouver, BC, Canada; ^6^Office of the Chief Medical Health Officer, Vancouver Coastal Health Authority, Vancouver, BC, Canada; ^7^School of Population and Public Health, University of British Columbia, Vancouver, BC, Canada

**Keywords:** climate change, extreme events, heat, messaging, smoke, wildfire

## Abstract

With the growing climate change crisis, public health agencies and practitioners must increasingly develop guidance documents addressing the public health risks and protective measures associated with multi-hazard events. Our Policy and Practice Review aims to assess current public health guidance and related messaging about co-exposure to wildfire smoke and extreme heat and recommend strengthened messaging to better protect people from these climate-sensitive hazards. We reviewed public health messaging published by governmental agencies between January 2013 and May 2023 in Canada and the United States. Publicly available resources were eligible if they discussed the co-occurrence of wildfire smoke and extreme heat and mentioned personal interventions (protective measures) to prevent exposure to either hazard. We reviewed local, regional, and national governmental agency messaging resources, such as online fact sheets and guidance documents. We assessed these resources according to four public health messaging themes, including (1) discussions around vulnerable groups and risk factors, (2) symptoms associated with these exposures, (3) health risks of each exposure individually, and (4) health risks from combined exposure. Additionally, we conducted a detailed assessment of current messaging about measures to mitigate exposure. We found 15 online public-facing resources that provided health messaging about co-exposure; however, only one discussed all four themes. We identified 21 distinct protective measures mentioned across the 15 resources. There is considerable variability and inconsistency regarding the types and level of detail across described protective measures. Of the identified 21 protective measures, nine may protect against both hazards simultaneously, suggesting opportunities to emphasize these particular messages to address both hazards together. More precise, complete, and coordinated public health messaging would protect against climate-sensitive health outcomes attributable to wildfire smoke and extreme heat co-exposures.

## Introduction

Increasing wildfire smoke and extreme heat events in North America are inextricably linked to climate change (1, 2), and exposure to each hazard has significant population health impacts (3–5). Moreover, data from Washington (6), Oregon (7), and California (8, 9) demonstrate that wildfire smoke and extreme heat often co-occur in western North America. Such phenomena are especially concerning in light of emerging epidemiologic evidence suggesting that the combined risk from co-exposure to wildfire smoke and extreme heat is greater than each health risk individually (9–11). Consequently, public health agencies and practitioners are increasingly challenged with developing and disseminating public health guidance that addresses the increasing risks of such multi-hazard events (12). Such multi-hazard events exacerbated by climate change also implicate multiple jurisdictions and even countries, simultaneously suggesting an urgent need for policies that foster coordinated messaging and related operations between multiple levels of government.

Public health policy encompasses a set of governmental measures involving legislation, rules, plans, and actions designed and implemented to attain public health objectives within a community or society (13). Developing and implementing appropriate public health guidance using effective messaging is widely recognized as an essential component of public health policy and practice (14). Research has shown that effective public health messages should aim to increase and clarify people’s understanding and perceptions of a health threat, their perceived susceptibility, and their perceived efficacy to take adequate preventive measures (15, 16). From an agency perspective, developing these public health messages must balance the need to communicate the threats and respective efficacies of different interventions publicly and accurately with the need to harmonize this messaging with current and evolving public policy and resources.

There are several practical challenges in developing effective public health guidance and related messaging in this context. First, different places and populations will vary regarding their health risks from exposure to wildfire smoke and extreme heat. Additionally, individual-level ability and local capacity to respond to wildfire smoke and extreme heat co-occurrence varies regionally. Thus, public health messaging at a national level provides more generalized guidance, occasionally with more specific guidance for at-risk populations. In contrast, local governmental agencies are better situated to develop and promote public health messages tailored to the local context, thus ensuring the information reaches at-risk or hard-to-reach populations. These complementary roles between national and local agencies present an opportunity for enhancing coordination, shaping more effective public health messaging around combined wildfire smoke and extreme heat, and ultimately advancing climate resilience (12, 17).

Recent evidence from Canada (18) and the US (19–21) highlights opportunities to improve the practice of public health messaging regarding wildfire smoke and extreme heat events. Evidence suggests a need for enhanced communication strategies and messaging to at-risk populations and inclusive, uniform, evidence-informed recommendations (18, 19). In WA, for example, evidence-informed interventions such as the use of high-efficiency particulate air (HEPA) filters and “do-it-yourself” (DIY) box fan filters, or the distribution of these resources by public health organizations, were among the least common personal or administrative interventions mentioned by local and state government agencies during the 2018 wildfire season (19). Communication gaps in public health guidance and messaging regarding extreme heat have also been observed in the US. A content analysis of local health department messaging in the US about extreme heat found gaps in mentioning or outreach for several at-risk populations (e.g., non-English speakers, specific medication use) and limited instances of translating public health messages for non-English speakers (20). Another review of national and local US agencies about extreme heat messaging found similar gaps regarding inconsistent mentioning of at-risk groups and limited messaging around measures to keep the indoor environment cool among those without air conditioning (21).

The demand and complexity of public health guidance and messaging during wildfire events are further complicated during extreme heat events. For instance, seeking shelter indoors is recommended as a personal intervention during wildfire smoke and extreme heat events. However, in specific settings, such as buildings without air conditioning or filtration systems, these evidence-based protective measures necessary to ensure cleaner indoor air or adequate cooling can pose conflicting challenges (22, 23). Buildings without air conditioning often rely on natural ventilation by opening doors or windows to keep the indoor air cool. However, minimizing smoke infiltration during wildfire events requires keeping windows and doors closed, trapping heat, and contributing to potentially dangerous indoor conditions during a co-occurring heat emergency.

This review assesses existing public health messaging from Canada and the US about the combined exposure and health risks of wildfire smoke, extreme heat, and personal interventions to reduce combined exposures. Based on this assessment, we provide recommendations for enhancing public health practice through more effective messaging related to these interconnected climate-sensitive hazards.

## Assessment of public health messaging

### Approach

Our assessment of current public health messaging focuses on public-facing government resources available online. We searched public health agency websites for information about mitigating health risks from co-exposure to wildfire smoke and extreme heat. Websites eligible for the assessment included local, provincial, or national agencies in Canada and local, state, and national agencies in the US. Regional attention was given to states in the western US (Washington, Oregon, and California) and provinces in western Canada (British Columbia, Alberta) because of the relatively significant impacts of wildfire smoke in these regions. All public-facing resources were identified using key search phrases in Google, including “wildfire smoke” AND [“extreme heat” or “heat wave”]. Available resources were eligible if a government agency published them, discussed co-exposure to wildfire smoke and extreme heat, suggested or mentioned protective health measures for either hazard, and published within the past decade (January 1, 2013, and May 31, 2023). We selected search results from the keyword search in Google for their eligibility by identifying the associated website indicated within the Google search result window and determining if it was marked as a government agency. We then reviewed these websites for eligibility based on the criteria indicated above.

An extreme heat event is typically defined as two or more consecutive days with outdoor temperature levels well above seasonal norms. However, the definition can vary widely between countries and even within a country. Therefore, in this review, we did not apply a strict criterion for defining an extreme heat event to establish eligibility for review.

Next, we reviewed and assessed each eligible resource according to the four themes outlined below. These themes were selected because they can inform people about health risks from co-exposure to wildfire smoke and extreme heat.

Themes assessed:

1. Vulnerable groups and risk factors: Individuals with one or more risk factors associated with greater exposure or risk factors for health effects from wildfire smoke and extreme heat exposure.
2. Symptoms for both exposures: A description of signs and symptoms of health effects associated with exposure to wildfire smoke and extreme heat.
3. Health risks of each hazard individually: A description of specific health risks or population health impacts associated with exposure to wildfire smoke and extreme heat separately.
4. Combined health risks: A description of the possibility of synergistic or interacting effects from co-exposure to wildfire smoke and extreme heat.

We note that themes two, three, and four are inherently interrelated. However, we chose to disaggregate these interrelated concepts because doing so enables us to assess potential inconsistencies and opportunities for improvement in public health guidance and messaging.

After reviewing and assessing each resource for content across these four themes, we conducted a more in-depth assessment of the different protective measures they described. Here, we reviewed each document for any guidance that could prompt individuals to take action to reduce their health risks from co-exposure to wildfire smoke and extreme heat. We continued our search of online resources until we reached saturation regarding the public health measures identified. Saturation in qualitative research refers to the point at which data collection and/or analysis is stopped because the same themes occur repeatedly with no new information being revealed (24).

Our assessment of protective measures for wildfire smoke, extreme heat, or co-exposure (Table 1) was meant to be inclusive, such that we captured established and emerging approaches. By emerging approaches, we refer to protective measures that occurred for the first time in the previous 2 years of health messaging included in this review. The evidence base supporting each measure was not reviewed, as this was beyond the scope of the assessment; however, all interventions recorded are informed by existing evidence.

**Table 1:** Protective measures identified and assessed in this review.^a^

Type of protective measure	Applicability of protective measure	Protective measure	Frequency (%) of measures mentioned across resources
Building	Wildfire Smoke	Keep windows and doors closed to limit smoke infiltration	10 (67%)
	Wildfire Smoke	Minimize other sources of indoor air pollution	7 (47%)
	Wildfire Smoke	Clean/filter air using a portable air cleaner	12 (80%)
	Wildfire Smoke	Clean/filter air with improvised (DIY) box fan devices	5 (33%)
	Extreme Heat	Stay cool indoors with timely use of windows/ blinds /curtains	5 (33%)
	Wildfire Smoke + Extreme Heat	Use air conditioning	15 (100%)
Individual	Wildfire Smoke	Go to a cleaner air center	9 (60%)
	Wildfire Smoke	Wear a protective face mask or respirator	9 (60%)
	Wildfire Smoke + Extreme Heat	Stay hydrated	11 (73%)
	Extreme Heat	Stay cool outdoors (e.g., in a shaded area, resting, misting)	5 (33%)
	Extreme Heat	Cool body with cool water or cool foods/clothing	8 (53%)
	Extreme Heat	Go to a cooling center	10 (67%)
	Wildfire Smoke + Extreme Heat	Limit outdoor and strenuous activity/exercise	11 (73%)
	Wildfire Smoke + Extreme Heat	Seek medical care, as needed	11 (73%)
	Wildfire Smoke + Extreme Heat	Seek support from friends or family	8 (53%)
	Wildfire Smoke + Extreme Heat	Check-in on others	7 (47%)
	Wildfire Smoke + Extreme Heat	Prioritize heat avoidance over wildfire smoke avoidance	6 (40%)
	Wildfire Smoke + Extreme Heat	Find or create an area with cleaner and cool air (indoors or outdoors)	6 (40%)
Access to information	Wildfire Smoke	Check air quality data and forecasts, air quality warning or advisory systems	10 (67%)
	Extreme Heat	Check temperature forecasts/ heat warning system	7 (47%)
	Wildfire Smoke + Extreme Heat	Install/review data from sensors to monitor local/household indoor and outdoor PM_2.5_ and temperatures	2 (13%)

a Protective measures indicated in this table are those identified in this review only. No specific measures were selected a priori to this review.

### Scoring for assessment of themes and protective measures

We developed and implemented an *ad hoc* scoring system to synthesize the reviewed resources, as described in Table 2. This approach allowed us to qualitatively assess the consistency of messaging and the completeness of each resource’s information. Individual scores were summed separately for the four themes (Thematic Messaging Score) and the protective measures (Protective Measures Score). Values of 0–2 were used to score Thematic Messaging. Since this review emphasizes messaging around protective measures, an additional value of 3 was only assigned to evaluate and score the “protective measures” identified in this review. Here, a score of 3 for protective measures was assigned if the information has the potential to substantively improve the clarity and precision of current public health messaging as it relates to protection against combined exposures.

**Table 2:** Scoring and color coding to visualize and synthesize themes and health protective measures.

Score^a^	Color	Interpretation	Assessment
0		Concept not mentioned	Themes and specific health protective measures
1		Concept mentioned but only partially addressed, i.e., information is incomplete or lacks clarity (i.e., ambiguous)	Themes and specific health protective measures
2		Concept mentioned and thoroughly addressed (i.e., information is complete and is clear)	Themes and specific health protective measures
3		Concept mentioned and thoroughly addressed, and the information addresses nuance for dealing with co-exposures to wildfire smoke and extreme heat	Specific health protective measures only

a Themes were assesses on a scale of 0 to 2 whereas protective measures were scored on a scale of 0 to 3.

After evaluating each resource, we assigned it a specific score (Table 2) for how well it addresses each protective measure. We then aggregated these scores for each protective measure, summing them across all the resources reviewed. We generated graphical displays to visualize potential opportunities to strengthen public health guidance and messaging from governmental agencies regarding co-exposure to wildfire smoke and extreme heat. We assessed all materials subjectively, relying on expert judgment from decades of collective public health and environmental agency experience and perspectives among co-authors.

### Assessment results

We identified and reviewed 15 online resources containing public health messaging about co-exposure to wildfire smoke and extreme heat—nine from Canada and six from the US (Table 3). One Canadian resource was nationally oriented; eight were created at British Columbia’s provincial or local levels. In contrast, half of the identified resources from the US were developed at the national level, including a webpage from the US Center for Disease Control and Prevention (CDC; *n* = 1) and a series of inter-agency wildfire smoke guidance documents from the US EPA (*n* = 2). The balance of US resources was from the California Department of Public Health (*n* = 1), the Missoula City-County Health Department in Montana (*n* = 1), and the San Joaquin Valley Air Pollution Control District in California (*n* = 1). We did not identify eligible resources for Washington State and Oregon for the review period (January 2013 to May 2023). Half of the reviewed resources were published in 2022 and 2023, suggesting governments increasingly recognize co-exposure to wildfire smoke and extreme heat as a multi-hazard public health issue.

**Table 3:** Characteristics of the identified resources included in this review.

Government agency, year of publication	Document type	Country	National, regional, or local	Interagency document^a^	Primary focus of resource^b^
Health Canada, 2021 (23)	Webpage or Factsheet: Extreme Heat and Wildfire Smoke	Canada	National	No	Both wildfire smoke and extreme heat
British Columbia Ministry of Emergency Management and Climate Readiness, 2022 (45)	Guidance Document: Extreme Heat	Canada	Regional	Yes	Extreme heat only
British Columbia Centre for Disease Control, 2022 (26)	Webpage and Factsheet: Extreme Heat and Wildfire Smoke	Canada	Regional	Yes	Both wildfire smoke and extreme heat
British Columbia Centre for Disease Control, 2023 (46)	Guidance Document: Extreme Heat	Canada	Regional	Yes	Extreme heat only
US Environmental Protection Agency, 2016 (29)	Guidance Document: Wildfire Smoke	US	National	Yes	Wildfire smoke only
US Environmental Protection Agency, 2019 (24)	Guidance Document: Wildfire Smoke (updated 2019)	US	National	Yes	Wildfire smoke only
California Department of Public Health, 2022 (51)	Guidance Document: Wildfire Smoke (updated in 2022)	US	Regional	No	Wildfire smoke only
Government of Alberta, 2023 (47)	Webpage or Factsheet: Extreme Heat	Canada	Regional	No	Extreme heat only
National Collaborating Centre for Environmental Health, 2018 (77)	Report: Wildfire Smoke	Canada	National	No	Wildfire smoke only
Vancouver Coastal Health, 2022 (54)	Webpage or Factsheet: Extreme Heat	Canada	Local	No	Extreme heat only
British Columbia Centre for Disease Control, 2014 (30)	Guidance Document: Wildfire Smoke	Canada	Regional	No	Wildfire smoke only
Missoula City-County Public Health Department, 2023 (52)	Webpage or Factsheet: Wildfire Smoke	US	Local	No	Wildfire smoke only
San Joaquin Valley Air Pollution Control District, 2022 (28)	Press Release: Extreme Heat and Wildfire Smoke	US	Local	No	Both wildfire smoke and extreme heat
British Columbia Housing (27)	Webpage or Factsheet: Extreme Heat and Wildfire Smoke	Canada	Regional	Yes	Both wildfire smoke and extreme heat
US Center for Disease Control and Prevention, 2022 (53)	Webpage or Factsheet: Wildfire Smoke	US	National	No	Wildfire smoke only

a Interagency resources are noted to help evaluate whether such documents provide more comprehensive public health messaging compared to resources developed without consultation of other agencies.

b While any identified agency resource was eligible if they discussed protective measures in the context of co-occurrence or co-exposure to wildfire smoke and extreme heat, we denote the focus of each resource to help distinguish the resources that were developed specifically to address co-exposures, and to evaluate whether such resources provide more clear and complete public health messaging.

We classified the identified resources into four categories, including guidance documents (*n* = 6), webpages or factsheets (*n* = 7), a press release (*n* = 1), and a report (*n* = 1). The reviewed guidance documents targeted public health agencies and partners, whereas webpages and factsheets targeted the general public. All the guidance documents and webpages or factsheets from the US focus on wildfire smoke, whereas most (78%) guidance documents and webpages or factsheets from Canada focus on extreme heat. For resources that focus on wildfire smoke, extreme heat is generally discussed as an additional stressor during a wildfire smoke event. In contrast, the extreme heat-focused resources discuss wildfire smoke as an additional stressor during extreme heat events. We found at least four resources explicitly motivated by addressing co-exposure to wildfire smoke and extreme heat; these include three webpages or factsheets from Canada (22, 26, 36) and one press release (35) from the US.

### Themes

#### Vulnerable groups and risk factors

All the included resources addressed vulnerable groups of people and risk factors (Table 4). These included intrinsic risk factors such as life stages (older adults, children, and teenagers), pregnancy, chronic health conditions (respiratory, cardiovascular, and psychiatric illnesses), and physical or mental limitations (limited mobility, people on medications that affect the body’s thermoregulation, substance use disorders). These also included extrinsic factors that increase risk, such as smoking, athletics, homelessness, living alone, working outdoors, living in remote communities, racialized minorities, low socioeconomic status, poor housing quality or living in high-rise apartment buildings, and people without air conditioning, air filtration, or health care access.

**Table 4:** Summary scores^a^ of themes for each resource included in the review.

Government agency, year of publication	Vulnerable groups and risk factors	Symptoms for both exposures	Health risks of each hazard individually	Combined health risks
Health Canada, 2022 (23)	2	1	0	0
British Columbia Ministry of Emergency Management and Climate Readiness, 2022 (24)	2	0	0	0
British Columbia Centre for Disease Control, 2022 (26)	2	2	2	2
British Columbia Centre for Disease Control, 2023 (46)	2	0	1	0
US Environmental Protection Agency, 2016^b^ (29)	2	1	2	0
US Environmental Protection Agency, 2019^b^ (24)	2	1	2	0
California Department of Public Health, 2022 (51)	2	0	1	0
Government of Alberta, 2023 (47)	2	1	0	0
National Collaborating Centre for Environmental Health, 2018 (77)	2	1	0	0
Vancouver Coastal Health, 2022 (54)	2	0	0	0
British Columbia Centre for Disease Control, 2014 (30)	2	0	0	2
Missoula City-County Public Health Department, 2023 (52)	2	0	0	0
San Joaquin Valley Air Pollution Control District, 2022 (28)	2	0	0	0
British Columbia Housing, 2022 (27)	2	2	1	0
US Center for Disease Control and Prevention, 2022 (53)	2	1	0	0

a Each theme was scored on a scale from 0 to 2 (refer to Table 2 for score descriptors).

b Series of guidance documents from the US Environmental Protection Agency originally published in 2008 that was updated in 2016 and in 2019.

Although we did not find a resource that comprehensively mentioned all of these risk factors, interagency guidance documents (23) and webpages and factsheets (26, 36) from the US Environmental Protection Agency (US EPA) and the British Columbia Center for Disease Control (BCCDC) mentioned at least seven or more of these groups. The most consistently mentioned susceptible and/or vulnerable groups included older populations, young children, pregnant people, outdoor workers, people with chronic health conditions such as asthma, chronic obstructive pulmonary disease, and cardiovascular disease, and those in homes without air conditioning. At-risk groups inconsistently highlighted were those with the following characteristics: unhoused, low socioeconomic status, different racial/ethnic minorities, non-English speaking, using medications that disrupt thermoregulation, living in poor-quality housing or high-rise apartments, with psychiatric illness or physical disabilities.

#### Symptoms for both exposures

Our review finds that distinctions of symptoms from wildfire smoke versus extreme heat are rarely described in public health messaging. Less than half (47%) of the resources mention or describe any symptoms of wildfire smoke and extreme heat exposure. Just two resources (26, 36) outline the different symptoms of these hazards.

#### Health risks of each hazard individually

Less than half (40%) of the resources mention or describe the known health risks from wildfire smoke or extreme heat events. Only three resources describe the distinct health effects of wildfire smoke and extreme heat within the same document. These three resources include guidance documents from the US EPA (23, 28) and a factsheet from the BCCDC (26). The BCCDC factsheet provides data that compares the relative health impacts on mortality from an extreme heat event and a wildfire smoke event (26). The US EPA’s wildfire smoke guidance document mentions illnesses associated with wildfire smoke. It provides descriptions of specific heat-related illnesses, such as heat stress, heat rash, heat cramps, and heat stroke.

#### Combined health risks

Only two identified resources (both from the BCCDC) address the potential for the combined risk from exposure to extreme heat and wildfire smoke to result in synergistic health effects (26, 33). A wildfire smoke guidance document produced by the BCCDC in 2014 writes, “Health risks may be compounded if heat waves and smoke occur concurrently as many of the same populations are vulnerable to both heat and smoke.” A factsheet by the BCCDC in 2022 writes, “Smoke and heat both put the human body under stress. Combined exposure may lead to more severe symptoms.”

#### Protective measures

**Figure 1:**
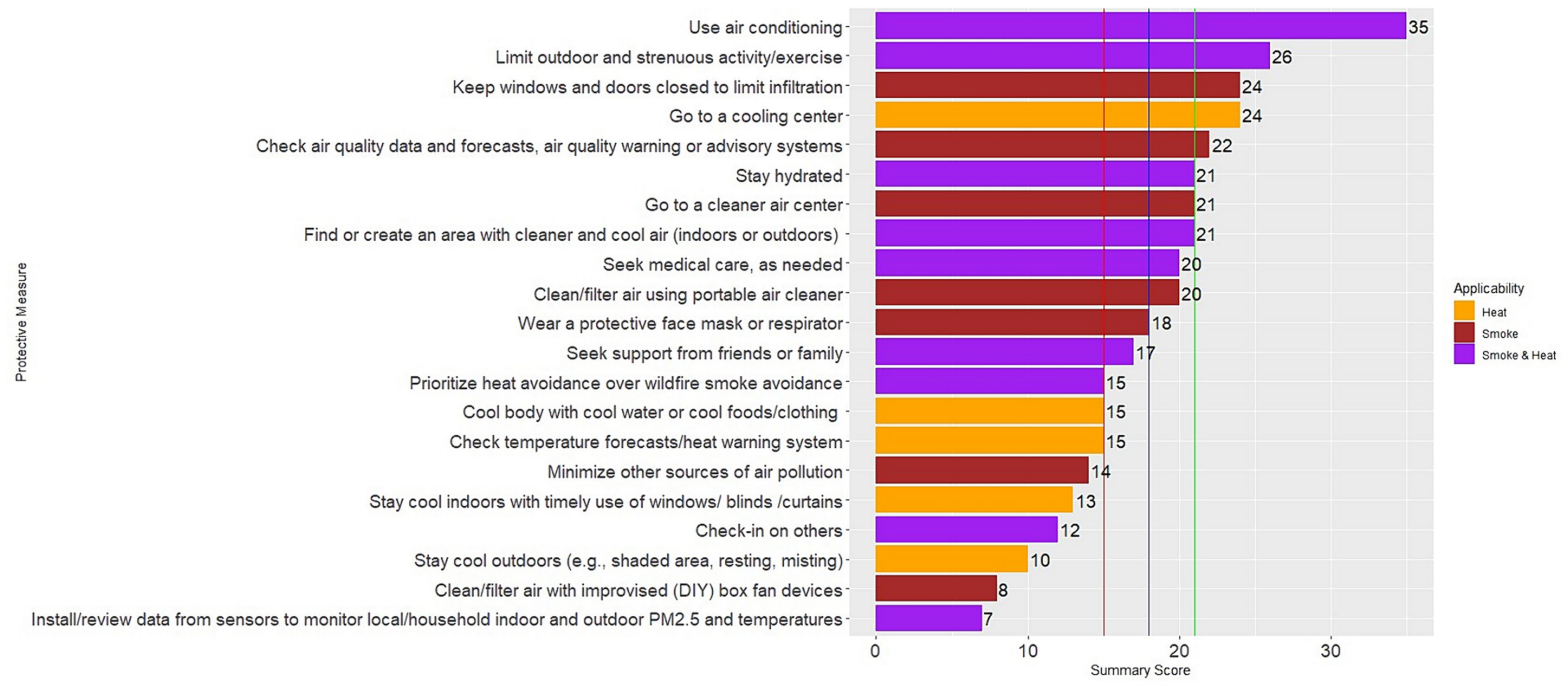
Summary score for different protective measures. Purple bars reflect measures that can be taken to mitigate co-exposure to wildfire smoke and extreme heat. Orange bars are measures specific to extreme heat exposure only. Brown bars are measures specific to wildfire smoke exposure only. Vertical lines are for the lower tertile (red line), median (blue line), and the upper tertile (green line) of the summary scores across all dimensions.

In our assessment of guidelines for protective measures, we identified and evaluated 21 interventions of interest. Figure 1 provides an overall summary of the scores for each protective measure, while Supplementary Table S1 details the scores assigned to each resource. To derive the summary scores for each protective measure, we summed the resource-specific scores for each protective measure, resulting in a mean score of 18 (range: 7 to 35) across all measures. The highest-ranking protective measures (upper third of distribution [>21]) represent the measures most consistently described across the resources. These high-ranking measures include using air conditioning, limiting outdoor or strenuous activities, going to a cooling center, closing windows and doors, or checking air quality warning/forecast systems. Other protective measures that ranked in the bottom tier of scores (lower third of distribution [<15]) include using a protective face mask or respirator, minimizing other sources of indoor air pollution, checking heat alert systems, using alternative methods of staying cool indoors aside from air conditioning (e.g., timely use of window coverings), staying cool outdoors (e.g., using shade-covered areas), using an improvised (DIY) box fan air filter, and using low-cost air sensors to monitor indoor/outdoor air quality or temperature.

We found nine protective measures that address co-exposure to wildfire smoke and extreme heat (Figure 1, purple bars). Identified protective measures that addressed co-exposures include using air conditioning, limiting outdoor or strenuous activities like exercise, finding or creating areas with both cleaner (e.g., filtration and limiting indoor air pollution sources) and cooler air indoors or outdoors, seeking medical care, seeking support from friends or family, prioritizing mitigation of heat exposure over wildfire smoke, checking in on others, staying hydrated, and using low-cost air sensors to monitor indoor/outdoor air quality and temperature. Three of these protective measures for mitigating co-exposure ranked in the bottom tier based on our assessment; these include prioritizing one hazard over the other (e.g., extreme heat over wildfire smoke), checking in on others, and using low-cost air sensors to monitor indoor and outdoor PM_2.5_ and temperature.

#### Actionable recommendations

We found that public health agency resources on wildfire smoke and heat co-occurrence are varied in their content, addressing a range of themes and protective measures, with substantial inconsistencies identified. We recommend improving public health messaging in several areas for the co-occurrence of wildfire smoke and extreme heat.

There are opportunities to improve messaging on symptoms and health effects of co-exposure to wildfire smoke and extreme heat. Communicating signs and symptoms for at-risk groups is crucial. Although both hazards can lead to similar acute health outcomes, we recommend messaging that better distinguishes their distinct biological effects and symptoms (38, 39). Extreme heat can result in various heat-related illnesses, characterized by symptoms like heavy sweating, a fast, weak pulse, and nausea (40). In contrast, wildfire smoke exposure leads to different symptoms, such as airway mucous production and wheezing or irritation (among others) caused by pulmonary inflammation and oxidative stress (38, 39). Clear communication about these distinguishing signs and symptoms can help individuals identify the specific hazard affecting their health, which can support decisions around the prioritization of reducing exposure to a specific hazard. In addition to self-management, this is practical information for the initial management of symptoms among vulnerable groups by the school or residential long-term care facilities staff.

Our review found limited and inconsistent communication about wildfire smoke and extreme heat’s distinct or overlapping health outcomes. Such information can help identify vulnerable populations and prioritize hazard protection based on individual risk factors. For example, individuals with a mental health disorder or mental illness are at greater risk of emergency department visits (41) or mortality (42, 43) during an extreme heat event. However, there is no evidence for such risks among this group due to exposure to wildfire smoke (although wildfires can negatively impact mental health in impacted areas (44)).

Another area of potential thematic improvement relates to communicating combined health risks from co-exposure to wildfire smoke and extreme heat. Discussing combined health risks may be limited due to the sparse epidemiologic evidence regarding the combined risk from co-exposure to wildfire smoke and extreme heat (7, 10, 11, 45). However, there is extensive evidence, across multiple systematic reviews (46, 47) and meta-analysis studies (48–50), supporting the hypothesis of more significant than additive health effects from co-exposure to extreme heat and ambient PM (i.e., not wildfire-specific). We identified just two examples of public health messaging around combined effects (26, 33), even though the existing evidence suggests there may be synergistic health effects from combined exposure to PM (PM2.5 and PM_10_) and extreme heat. Because exposure events may overlap, a greater emphasis on public health messaging about the combined mortality and morbidity risks from co-exposure to wildfire smoke and extreme heat is warranted. Public health messaging would also benefit from epidemiological research clarifying sub-population susceptibility to these combined exposures (e.g., prenatal co-exposure health effects).

Several Canadian resources recommend prioritizing reducing extreme heat over wildfire smoke exposure when effectively managing both is impractical (25–27, 30, 36). This prioritization issue becomes relevant because some of the most common guidance for mitigating exposure to wildfire smoke is to stay indoors with closed windows and doors. This protective measure can lead to dangerous indoor temperatures during extreme heat events, especially in regions without widespread air conditioning. However, it is essential to remember that the risk from extreme heat is higher in areas with less heat exposure history (51, 52), and even within regions, there can be transient seasonal heat acclimatization (29). Thus, it is plausible that wildfire smoke may pose a greater risk than heat exposure later on in the hotter time of the year. Therefore, widespread messages prioritizing heat or smoke may lack nuanced considerations of individual, household, and regional contexts. Blanket statements favoring one over the other need more unambiguous evidence and require careful assessment of at-risk groups, regional acclimatization, and seasonal factors for practical guidance.

Public health messaging at the local level is also needed to provide more flexible and nuanced guidance for at-risk populations facing potentially conflicting actions when protecting against wildfire smoke and extreme heat. Such guidance is urgent for communities susceptible to the urban heat island effect, such as those in high-density urban housing and those without access to air conditioning, air filtration, or cooling centers. We identified several regional and national-level resources (23, 25–30, 32–34, 37) offering more nuanced advice like using thermal curtains to reduce indoor heating, opening windows when the smoke dissipates for ventilation and air cleaning, and night-time window opening for cooling.

The US and Canada have separate heat warning systems, and varied systems exist within each country. Only 40% of reviewed resources suggested checking these systems. In contrast, most resources (73%) mentioned checking wildfire smoke or air quality warning systems. Among the US resources reviewed, only the US CDC recommended checking a heat warning system (37). Improving public health messaging should address this gap and motivate the integration of heat warnings into existing wildfire smoke systems. Weather-related warning systems are already structured as multi-hazard alerting systems and offer a model for integrating multiple hazards into publicly available warning systems. A multi-hazard warning system that explicitly integrates wildfire smoke and heat co-occurrence is worth considering as we learn more about the links between climate change and health and the demand for more effective public health communication strategies in North America.

##### Protective measures with possible co-benefits

Messaging on low-cost air quality sensors received the lowest score among protective measures. The California Department of Public Health recommends using low-cost air quality sensor networks to aid communities and households in understanding and responding to wildfire smoke, citing examples like PurpleAir’s real-time data (29). These sensors also measure temperature and humidity, making them valuable for monitoring extreme heat and wildfire smoke co-exposure and guiding health behaviors. However, there are caveats. Most sensors lack calibrated PM_2.5_ values and appropriate time averaging for health-based messaging. The US EPA’s Fire and Smoke Map and the University of Northern British Columbia AQMap do correct PurpleAir sensor data for PM_2.5_ data visualization for the US and Canada, respectively. These data visualization tools also use more appropriate averaging times of corrected PM_2.5_ concentrations and relate these back air quality indices for health communication needs. Since these tools focus on outdoor air quality, they can support individual and household decisions like going outside or airing out homes. Thus, recommendations on low-cost air quality sensors should direct users to online tools whose measures and displays can map onto public health recommendations for various exposures.

We also considered monitoring indoor temperature using thermostats or thermometers, mentioned in only three resources (26, 30, 32), as a low-cost approach complementary to low-cost air quality sensors. This information can help individuals determine when it is safe to open windows to cool or ventilate their homes, benefiting susceptible or vulnerable populations without air conditioning.

Air conditioning or portable HEPA-filtered air cleaners were commonly recommended protective measures. On the other hand, do-it-yourself (DIY) box fan air filters were mentioned in only four resources (23, 26, 29, 37). DIY box fan filters are shown to enhance protection during wildfire smoke events by improving clean air delivery (53). A recent field study found that window-mounted DIY box fan filters improved air exchange and filtration, reducing indoor PM_2.5_ levels and thermal discomfort (54). Public health messaging should promote DIY box fan filters for low-resource households, especially those with chronic health conditions who cannot install air conditioning or air cleaners. Further field studies on window-mounted DIY air cleaners are warranted to evaluate their ability to reduce indoor PM levels and enhance thermal safety during wildfire smoke and extreme heat episodes.

Addressing co-exposure indoors also underscores the added challenges associated with significant social (55–57) and regional (58) disparities in North America, particularly regarding access to some of the most effective protective measures, such as air conditioning equipped with filtration and portable air cleaners. Notably, an analysis of data from 115 US metropolitan statistical areas reveals a higher prevalence of air conditioning absence among more socially vulnerable populations, particularly those residing in the urban core as opposed to their suburban counterparts (55). Disparities in air conditioning access have also been identified along racial lines (57) and concerning renter-owner occupancy in the US (59). Agencies must be aware that public health messaging narrowly emphasizing measures like conventional air conditioning with filtration may inadvertently overlook populations who lack the necessary resources and who may already be grappling with multiple risk factors. Messaging around keeping indoor spaces cool using alternative means than air conditioning, such as timely opening of windows in the cooler evenings or using curtains or cardboard on windows to block heat from the sun, ranked in the lowest tier of protective measures based on our assessment. Moreover, the least mentioned at-risk groups across the reviewed resources were the unhoused, low socioeconomic status, marginalized racial/ethnic minority groups, non-English speaking, and those living in poor-quality housing or high-rise apartments. Our findings and others (18, 20, 60–62) show an urgent need for more consistent and targeted public health messaging and outreach for populations with intersecting risk factors.

Cooling centers for the public may provide cleaner indoor air but are contingent on factors like facility manager awareness of wildfire smoke impacts, building capabilities, and air conditioning. Furthermore, their effectiveness in mitigating wildfire smoke exposure is unknown mainly because there is sparse data on their usage among at-risk groups and whether indoor air pollution levels during wildfire smoke events are lower at these cleaner air facilities. Cleaner indoor air centers may also offer cooler air. Local officials should consider barriers (e.g., strong preference to stay at home, accessibility) and facilitators (e.g., effective outreach) when recommending these centers for at-risk populations (32).

British Columbia government agencies are working to enhance public health messaging and awareness about cooling and cleaner air centers. The Vancouver Coastal Health Authority (VCH), a British Columbia-based public health agency, includes information on these centers in their messaging regarding combined wildfire smoke and extreme heat (32), including their locations, contact information, and designated uses. VCH is also piloting low-cost air sensor networks in informal, cleaner indoor air centers to understand their effectiveness in preventing wildfire smoke exposures. The British Columbia government is inventorying and mapping publicly available centers, including facility-specific capacities, through an online portal for use by local officials. Adapting such activities can strengthen local public health messaging and response during wildfire smoke and extreme heat co-occurrence in western North America and likely other regions.

Checking on others was infrequently mentioned, especially in wildfire-focused resources (Figure 1). While research highlights the importance of individual vulnerability during extreme heat events at home or alone (63–69), emphasizing this simple message for wildfire smoke is warranted. Prioritizing checking on others can benefit vulnerable communities during wildfire smoke and extreme heat events (70).

##### Local planning for the co-occurrence of wildfire smoke and extreme heat

Regions face unique climatic and population vulnerabilities and adaptive capacities, requiring tailored messaging and preseason preparedness. Planning for the co-occurrence of wildfire smoke and extreme heat would support the development of tailored public health messaging. The Vancouver Emergency Management Agency (VEMA) Extreme Heat and Wildfire Smoke Plan (71) offers one model for addressing these co-occurring hazards. The VEMA plan is being shared with other local, regional, and indigenous governments in British Columbia and is being adapted to suit the local context.

## Discussion

Ours is the first study to review and assess public health agency messaging on combined wildfire smoke and extreme heat exposure. Our findings of incomplete and inconsistent public health-related messaging across agencies are supported by similar gap assessments specific to wildfire smoke (18, 19) and extreme heat (21) public health messaging. Notably, only a small subset of the identified resources were designed to address co-exposure to wildfire smoke and extreme heat. At the same time, the balance was oriented around a specific hazard (e.g., wildfire smoke) and not intending to address their co-occurrence fully. The growing direct (heat) and indirect (wildfire smoke) climate change impacts across North America and the overlapping seasonal occurrence of wildfire smoke and extreme heat implies that public health agency guidance will need to emphasize that these hazards are occurring together more often and that messaging around protective measures for this emerging situation is warranted.

Based on our findings, we recommend further investment in clear, complete, and coordinated public health guidance and messaging by agencies across government levels to optimize protection against health risks from combined wildfire smoke and extreme heat. For example, more clarity and complete information regarding symptoms and at-risk populations are needed. We found that listed symptoms are frequently lumped together (regardless of whether they are from excessive heat or wildfire smoke exposure), or only one set of symptoms is listed for a particular exposure (e.g., symptoms from smoke exposure only with no description of symptoms from heat). Some key at-risk populations are infrequently described (e.g., unhoused and those with psychiatric disorders), so we recommend more careful consideration of at-risk populations for health messaging. Enhanced planning and coordination of public health messaging between different levels of government is also warranted. For example, national agencies can only highlight various at-risk/sensitive sub-groups. In contrast, state or local agencies must leverage that information to ensure that messaging about health protective measures appropriately reaches the at-risk populations within their jurisdiction.

Review and assessment of the eligible resources reveals notable differences between the US and Canada. In Canada, guidance documents and webpages were more frequently from local or provincial agencies, while US guidance documents and webpages were more frequently from national public health agencies (e.g., US EPA or CDC). Website-or factsheet-based health messaging from local health agencies in Canada tended to mirror the messaging from provincial guidance documents and websites/factsheets (e.g., BCCDC). In contrast, local health agency messaging in the US tended to mirror the messaging from national-level government guidance documents (e.g., US EPA). While it is unclear what the implications are for this difference, it does suggest the possibility that local agencies between the two countries take cues from different levels of government when developing health messaging.

Additionally, Canadian resources were heat-focused more often, while US resources were wildfire-smoke-focused. Regarding resources specifically designed to address co-exposures, three of these four resources were from Canada, and the only US-based resource was a press release from a local air quality agency. These findings suggest possible differences in perceived climate-sensitive health risks between the countries without a clear rationale for this discrepancy.

There were also notable themes between the resources specifically designed to address co-exposure to extreme heat and wildfire smoke. In Canada, the three resources fitting this category tended to suggest that extreme heat is generally more dangerous than wildfire smoke. Thus, the prevention of heat exposure should be prioritized over the prevention of wildfire smoke. As noted in our recommendations, this is a contentious claim based on regional differences in acclimatization and at-risk populations. Moreover, given the emerging evidence of synergistic health effects of extreme heat and wildfire smoke, it is plausible that the threshold for adverse health effects from heat exposure depends on wildfire smoke exposure levels and vice versa. The adverse health effects of wildfire PM_2.5_ (72) and temperature (73) may also be non-linear, with vastly different exposure-response curves between these exposures. Given uncertainties and complexities in the epidemiological data, more conclusive epidemiological evidence focused on co-exposure scenarios in varied geographic regions is needed to provide appropriately nuanced guidance for prioritizing one exposure over the other.

Our approach and findings have limitations. First, this work was not a systematic review of specific research databases using predefined search terms. Therefore, the protective measures discussed do not represent the breadth of measures in the peer-reviewed scientific literature. It is possible that insight can be gained by extending this search into the peer-reviewed literature, which we recommend as a next step to enhancing public health messaging. Additionally, we did not formally evaluate and synthesize the evidence base for protective measures for dealing with co-exposures, as this was beyond the scope of the review. It is plausible that recommendations regarding health messaging for co-exposures may be ineffective. Research is warranted to generate evidence for effective protective measures to reduce co-exposure to extreme heat and wildfire smoke. We reviewed public health agency messaging until saturation and did not explicitly review tribal/First Nations’ governmental resources. Therefore, future assessments should work with tribal/First Nations to learn about protective measures that may exist beyond those assessed in this review. Additionally, this review did not address smoke attributable to agricultural or prescribed burns, which is a limitation.

## Conclusion

Governmental agencies responsible for communicating during situations where wildfire smoke and extreme heat coincide have a unique opportunity to navigate the intricacies of this scenario with effective coordination and nuance. Providing more precise, consistent, and timely information regarding the health implications of simultaneous exposure to wildfire smoke and extreme heat and guidance on protective measures can significantly enhance public health communication in our changing climate.

## Author contributions

EC: Conceptualization, Data curation, Funding acquisition, Investigation, Methodology, Project administration, Visualization, Writing – original draft. SS: Resources, Writing – review & editing. EM: Writing – review & editing. JY: Conceptualization, Writing – review & editing. EB: Writing – review & editing. MS: Writing – review & editing. SH: Conceptualization, Funding acquisition, Supervision, Writing – review & editing.
