# Supplementary material for: Climate change and health: rethinking public health messaging for wildfire smoke and extreme heat co-exposures
Source: Front Public Health. 2024 Mar 25;12:1324662. doi: 10.3389/fpubh.2024.1324662 (PMC10999651; doi:10.3389/fpubh.2024.1324662)
Supplement: Supplementary file 1 [file Table_1.DOCX]

# **Supplementary Materials**

| **Table S1. Protective measure scores for each resource included in this review** | | | | | | | | | | | | | | | | | | | | | |
| --- | --- | --- | --- | --- | --- | --- | --- | --- | --- | --- | --- | --- | --- | --- | --- | --- | --- | --- | --- | --- | --- |
|  | **Wildfire Smoke Only** | | | | | | | **Extreme Heat Only** | | | | | **Wildfire Smoke + Extreme Heat** | | | | | | | | |
| **Government Agency, Year of Publication** | **Keep windows and doors closed to limit infiltration** | **Minimize other sources of air pollution** | **Check Air Quality Warning System** | **Use a DIY Box Fan Air Purifier** | **Go to a Clean Air Center** | **Use air Filtration or Commercial Air Purifier** | **Use a Face Mask** | **Staying cool indoors (timely use of windows/ blinds /curtains)** | **Check heat warning system** | **Staying Cool Outdoors (shaded area, misting)** | **Go to a Cooling Center** | **Cooling body with cool water or cool foods/clothing** | **Stay Hydrated** | **Limit outdoor and/or strenuous activity/exercise** | **Seek Medical Care** | **Seek support from friends or family** | **Check in on others** | **Prioritize Heat over Wildfire Smoke** | **Find areas with clean and cool air (indoors or outdoors)** | **Air Conditioning Usage** | **Sensors to Monitor Indoor/Outdoor Temperature or Air Quality** |
| Health Canada (2021)^a^ | 2 | 2 | 2 | 0 | 2 | 1 | 2 | 0 | 2 | 0 | 2 | 0 | 2 | 2 | 2 | 0 | 2 | 2 | 2 | 3 | 0 |
| BC EMCR (2022)^b^ | 0 | 0 | 1 | 0 | 0 | 0 | 0 | 3 | 2 | 2 | 2 | 2 | 2 | 2 | 2 | 2 | 2 | 2 | 0 | 2 | 0 |
| BCCDC (2022)^c^ | 2 | 0 | 2 | 2 | 2 | 2 | 2 | 0 | 2 | 0 | 2 | 0 | 1 | 0 | 3 | 3 | 0 | 2 | 2 | 2 | 0 |
| BCCDC (2023)^d^ | 0 | 0 | 0 | 0 | 0 | 0 | 0 | 3 | 3 | 2 | 2 | 2 | 2 | 1 | 1 | 2 | 2 | 2 | 1 | 2 | 1 |
| US EPA (2016)^e^ | 3 | 2 | 3 | 0 | 2 | 2 | 2 | 0 | 0 | 2 | 1 | 2 | 2 | 2 | 2 | 2 | 0 | 0 | 0 | 3 | 0 |
| US EPA (2019)^f^ | 3 | 2 | 3 | 2 | 2 | 2 | 2 | 1 | 0 | 2 | 2 | 2 | 2 | 2 | 2 | 2 | 0 | 1 | 2 | 3 | 2 |
| CDPH (2022)^g^ | 2 | 2 | 2 | 2 | 2 | 2 | 2 | 0 | 0 | 0 | 2 | 0 | 0 | 2 | 2 | 2 | 0 | 0 | 2 | 3 | 2 |
| Alberta (2023)^h^ | 0 | 0 | 1 | 0 | 0 | 0 | 0 | 2 | 2 | 2 | 2 | 2 | 2 | 2 | 2 | 2 | 2 | 2 | 1 | 2 | 1 |
| NCCEH (2018)^i^ | 2 | 0 | 2 | 0 | 2 | 1 | 2 | 0 | 0 | 0 | 0 | 0 | 0 | 2 | 0 | 0 | 0 | 0 | 1 | 1 | 0 |
| VCH (2022)^j^ | 0 | 0 | 0 | 0 | 3 | 1 | 0 | 2 | 2 | 0 | 3 | 2 | 2 | 2 | 2 | 0 | 2 | 0 | 0 | 2 | 1 |
| BCCDC (2014)^k^ | 2 | 2 | 2 | 0 | 2 | 2 | 2 | 0 | 0 | 0 | 0 | 0 | 0 | 2 | 0 | 0 | 0 | 0 | 2 | 3 | 0 |
| MCCPHD^l^ | 3 | 2 | 0 | 0 | 0 | 2 | 2 | 1 | 0 | 0 | 0 | 0 | 2 | 2 | 0 | 2 | 0 | 1 | 2 | 3 | 0 |
| Valley Air District (2022)^m^ | 0 | 0 | 2 | 0 | 0 | 1 | 0 | 0 | 0 | 0 | 2 | 0 | 2 | 1 | 0 | 0 | 2 | 0 | 2 | 2 | 0 |
| BC Housing^n^ | 3 | 2 | 0 | 0 | 2 | 2 | 0 | 1 | 0 | 0 | 2 | 3 | 2 | 2 | 2 | 0 | 0 | 2 | 2 | 2 | 0 |
| US CDC (2022)^o^ | 2 | 0 | 2 | 2 | 2 | 2 | 2 | 0 | 2 | 0 | 2 | 0 | 0 | 2 | 0 | 0 | 0 | 1 | 2 | 2 | 0 |

^a^Health Canada. Wildfire smoke 101: Combined wildfire smoke and heat. (2021) https://www.canada.ca/en/health-canada/services/publications/healthy-living/combine-wildfire-smoke-heat.html [Accessed April 24, 2023]

^b^BCEMCR. Be prepared for extreme heat - Province of British Columbia. (2022) https://www2.gov.bc.ca/gov/content/safety/emergency-management/preparedbc/know-your-hazards/severe-weather/extreme-heat [Accessed April 24, 2023]

^c^BCCDC. BCCDC_WildFire_FactSheet_HotWeather.pdf. (2022) http://www.bccdc.ca/resource-gallery/Documents/Guidelines%20and%20Forms/Guidelines%20and%20Manuals/Health-Environment/BCCDC_WildFire_FactSheet_HotWeather.pdf [Accessed April 24, 2023]

^d^BCCDC. Provincial-Heat-Alerting-Response-System.pdf. (2023) http://www.bccdc.ca/resource-gallery/Documents/Guidelines%20and%20Forms/Guidelines%20and%20Manuals/Health-Environment/Provincial-Heat-Alerting-Response-System.pdf [Accessed April 24, 2023]

^e^US EPA. Wildfire Smoke A Guide for Public Health Officials Revised May 2016. (2016). https://stacks.cdc.gov/view/cdc/80620

^f^US EPA. Wildfire Smoke A Guide for Public Health Officials Revised 2019. (2019).

^g^CDPH. Wildfire Smoke Considerations for California’s Public Health Officials. (2022). https://www.cdph.ca.gov/Programs/EPO/CDPH%20Document%20Library/EOM%20Documents/Wildfire-Smoke-Considerations-CA-PHO_08-2022.pdf

^h^Alberta. Extreme heat. (2023) https://www.alberta.ca/extreme-heat.aspx [Accessed April 25, 2023]

^i^NCCEH. PUBLIC HEALTH RESPONSES TO WILDFIRE SMOKE EVENTS. (2018). <https://ccnse.ca/sites/default/files/Responding%20to%20Wildfire%20Smoke%20Events%20EN.pdf>

^j^VCH. Extreme heat | Vancouver Coastal Health. (2022) https://www.vch.ca/en/extreme-heat [Accessed April 25, 2023]

^k^BCCDC, Elliott C. Guidance for BC Public Health Decision Makers During Wildfire Smoke Events. BCCDC (2014). http://www.bccdc.ca/resource-gallery/Documents/Guidelines%20and%20Forms/Guidelines%20and%20Manuals/Health-Environment/WFSG_BC_guidance_2014_09_03trs.pdf [Accessed April 25, 2023]

^l^MCCPHD. Wildfire Smoke Protective Measures | Missoula County, MT. (2023) https://www.missoulacounty.us/government/health/health-department/home-environment/air-quality/wildfire-smoke-protective-measures [Accessed April 25, 2023]

^m^Valley Air. Extreme heat and wildfire smoke expected Labor Day weekend | Valley Air District. (2022) https://ww2.valleyair.orghttps://ww2.valleyair.org/news-outreach-and-education/news/extreme-heat-and-wildfire-smoke-expected-labor-day-weekend/ [Accessed April 25, 2023]

^n^BC Housing. Extreme Heat and Wildfire Smoke Health Impacts | BC Housing. https://www.bchousing.org/projects-partners/extreme-heat/health-impacts [Accessed April 25, 2023]

^o^US CDC. Public Health Strategies to Reduce Exposure to Wildfire Smoke during the COVID-19 Pandemic | CDC. (2022) https://www.cdc.gov/disasters/covid-19/reduce_exposure_to_wildfire_smoke_covid-19.html [Accessed April 26, 2023]
